# A Gyroid TPMS-based machine learning framework for rapid prediction of cancellous bone mechanical properties

**DOI:** 10.3389/fbioe.2026.1870920

**Published:** 2026-09-15

**Authors:** Xinzhe Chen, Jiqing Chen, Fengchong Lan

**Affiliations:** South China University of Technology, School of Mechanical & Automotive Engineering, Guangzhou, China

**Keywords:** cancellous bone, machine learning, mechanical properties, parametric modeling, TPMS

## Abstract

**Introduction:**

High-fidelity finite-element analysis of cancellous bone is computationally expensive, particularly when large parameter spaces must be explored. This study developed a rapid surrogate-modeling framework integrating parametric Gyroid modeling, finite-element analysis, and machine learning to predict the apparent mechanical properties of cancellous-bone models.

**Methods:**

Trabecular tissue Young’s modulus (*E*_t_), tissue failure strain (*ε*_t_), bone volume fraction (*BV/TV*), and degree of anisotropy (*DA*) were used as inputs. A database of 200 parameter combinations was generated using Latin hypercube sampling, with apparent Young’s modulus (*E*_c_) and failure stress (*σ*_c_) obtained from finite-element simulations as separate outputs. Multiple linear regression, quadratic polynomial regression, support vector regression, Kriging, and a backpropagation neural network were evaluated using nested five-fold cross-validation.

**Results:**

The finite-element model reproduced published uniaxial compression responses. BPNN achieved the best prediction of *E*_c_ (R_2_ = 0.9993 ± 0.0003, NRMSE = 0.0256 ± 0.0027, MAPE = 1.68 ± 0.38%), whereas SVR performed best for *σ*_c_ (R_2_ = 0.9987 ± 0.0004, NRMSE = 0.0352 ± 0.0030, MAPE = 2.23 ± 0.45%). Response-surface analysis showed that *E*_t_ and *BV/TV* primarily governed apparent stiffness, while failure stress was additionally sensitive to *ε*_t_.

**Discussion:**

The proposed framework provides an accurate and computationally efficient approach for rapid prediction, parameter screening, response-surface analysis, and structural design exploration within the investigated Gyroid model space.

## 1 Introduction

Cancellous bone is a highly porous component of the skeletal system that is predominantly distributed in the epiphyseal and metaphyseal regions of long bones and within vertebral bodies. Its interconnected trabecular network contributes to load bearing, energy absorption, and metabolic regulation, while its deterioration is closely associated with fragility fractures in cancellous-bone-rich regions (Nazarian et al., 2006; De, 2024). The apparent mechanical properties of cancellous bone vary considerably among individuals and anatomical sites because they are jointly determined by tissue-level material properties and mesoscale architecture (Ogurkowska and Blaszczyk, 2020). At the mesoscale, trabeculae form an interconnected and directionally organized network that adapts to the local mechanical environment, resulting in pronounced structural anisotropy (Wolff, 1893). Characterizing the relationships between these tissue and structural features and the apparent mechanical response is therefore important for biomechanical assessment and the design of bone-mimicking porous structures.

Apparent density, ash density, and bone mineral density have been used to estimate the stiffness and strength of cancellous bone. Numerous empirical relationships have been established between density-related parameters and apparent Young’s modulus (Table 1), including the power-law relationship proposed by Carter and Hayes (Carter and Hayes, 1976). These equations were all derived by fitting experimental results for cancellous bone material; however, they cannot explain the differences in mechanical properties observed among samples with similar densities. Tissue-level measurements have demonstrated substantial variation in trabecular Young’s modulus (Wu et al., 2018), while differences in bone volume fraction, connectivity, and anisotropy can further alter the apparent response. Consequently, densitometric parameters alone are insufficient to completely characterize the mechanical behavior of cancellous bone (Alberich Bayarri, 2010).

**TABLE 1:** Empirical relationships between bone density and apparent Young’s modulus.

Bone type	Apparent Young’s modulus relationship $E_{\text{c}}$ (GPa)	References
Pooled	$E_{\text{c}}=3.79{\dot{\varepsilon }}^{0.06}\rho_{\text{app}}^{3}$	Carter and Hayes (1976)
Femur	$E_{\text{c}}=10.5\rho_{\text{ash}}^{2.29}$	Keller (1994)
Femoral head	$E_{\text{c}}=0.573\rho_{\text{app}}-0.0094$	Li and Aspden (2010)
Proximal femur	$E_{\text{c}}=7.541\left(BV/TV\right)-0.637$	Ciarelli et al. (2009)
Distal femur	$E_{\text{c}}=10.88\rho_{\text{ash}}^{1.61}$	Kaneko et al. (2004)

$E_{\text{c}}$ is the Young’s modulus of cancellous bone, $\dot{\varepsilon }$ is the strain rate, $\rho_{\text{app}}$ is the apparent density, $\rho_{\text{ash}}$ is the ash density, and BV/TV is the bone volume fraction.

The advancement of micro-computed tomography (micro-CT) and computational simulation techniques has yielded powerful tools for the detailed modeling and mechanical characterization of trabecular bone. Recent studies have used high-resolution micro-CT scans to construct mesoscale finite element models of trabecular bone, which are then used to analyze the relationship between apparent mechanical properties and mesoscale structure, often with validation against experimental compression data (Hambli, 2013; Megias et al., 2022; Belda et al., 2020; Vercher-Martínez et al., 2024). However, owing to the intricate geometry of trabecular bone, its mesoscale finite element models often contain millions of elements. These modeling and computational processes are therefore time-consuming and resource-intensive, highlighting the need in clinical practice for more rapid and straightforward assessment methods.

Parametric modeling provides an alternative means of constructing controllable cancellous-bone-like structures. Existing approaches have represented trabecular architecture using periodic honeycomb cells, diamond lattices, or stochastic strut networks (Goda and Ganghoffer, 2015; Yau et al., 2024; Yamada et al., 2025). Although these models facilitate systematic control of porosity and characteristic dimensions, strut- and plate-based unit cells cannot fully reproduce the continuous curvature of native trabecular surfaces. Triply periodic minimal surfaces (TPMS) offer smooth geometry, continuous pore interconnectivity, and reduced geometric discontinuities. In particular, Gyroid structures have demonstrated morphological and mechanical characteristics resembling those of cancellous bone (Huang et al., 2020). Nevertheless, most parametric porous models primarily investigate porosity, whereas the combined effects of bone volume fraction, structural anisotropy, tissue stiffness, and tissue failure properties remain less systematically characterized.

Machine-learning-based surrogate models offer a means of replacing repeated finite-element simulations once a representative simulation database has been established. Neural networks and other regression algorithms have been applied to biomechanical problems involving scaffold properties, soft-tissue response, bone remodeling, and load estimation (Garijo et al., 2017; Pellicer-Valero et al., 2020; Reddy et al., 2021; Pais et al., 2023). However, the predictive performance of a surrogate model depends on the complexity of the underlying parameter-response relationship, the size and distribution of the training database, and the validation strategy. In a moderate-sized simulation database with only a few input variables, a complex neural network is not necessarily superior to simpler regression methods. A reliable assessment therefore requires comparison with appropriate linear, polynomial, kernel-based, and probabilistic models, together with a validation framework that separates hyperparameter optimization from performance evaluation.

Accordingly, this study develops a computational framework integrating parametric Gyroid modeling, finite-element analysis, and regression-based surrogate modeling. Trabecular tissue Young’s modulus $E_{\text{t}}$ , tissue failure strain $\varepsilon_{\text{t}}$ , bone volume fraction BV/TV , and degree of anisotropy DA were selected as inputs, while the finite-element-derived apparent Young’s modulus $E_{\text{c}}$ and failure stress $\sigma_{\text{c}}$ were treated as separate outputs. A total of 200 parameter combinations were generated using Latin hypercube sampling. Multiple linear regression, quadratic polynomial regression, support vector regression, Kriging, and a backpropagation neural network were evaluated using identical nested five-fold cross-validation partitions. The optimal algorithm was selected separately for each mechanical response, and the final models were used to construct response surfaces describing the combined effects of tissue and structural parameters. The computational costs of geometry generation, finite-element modeling, numerical solution, model training, and prediction were also quantified.

The proposed method enables rapid prediction of finite-element responses within the sampled parameter range and can support parameter screening, sensitivity analysis, response-surface visualization, and structural design optimization. By combining controllable Gyroid models with systematic model comparison and rigorous validation, the study provides a clear and reproducible way to examine how tissue properties and mesoscale structure jointly affect the apparent mechanical behavior of cancellous bone.

## 2 Materials and methods

### 2.1 Gyroid-based parametric modeling of cancellous bone

As shown in Figure 1, cancellous bone is a porous material with pore sizes of 0.2 ∼ 2 mm and a highly interconnected structure that facilitates the supply and transport of nutrients. Wolff’s law (Wolff, 1893) states that the local mechanical stress state directly governs the processes of bone growth, resorption, and remodeling. Variations in mechanical stress modulate a dynamic equilibrium between osteoblast and osteoclast activity, with increased stress promoting bone formation and reduced stress accelerating resorption. Consequently, the porosity of cancellous bone varies significantly, ranging from 40% to 84% (Green et al., 2011; Perilli et al., 2008; Wu et al., 2013; Greenwood et al., 2015; Hambli, 2013) both among individuals and across different anatomical sites within a single person. The skeletal system continuously adapts by aligning its trabeculae along the principal stress trajectories. In femoral cancellous bone, this anisotropy is particularly pronounced, manifesting morphologically as irregular, elliptical structures whose major axes align with the principal load-bearing directions. This structural arrangement results in significant anisotropy in the mechanical response of cancellous bone, making its load-bearing capacity highly dependent on orientation.

**FIGURE 1:**
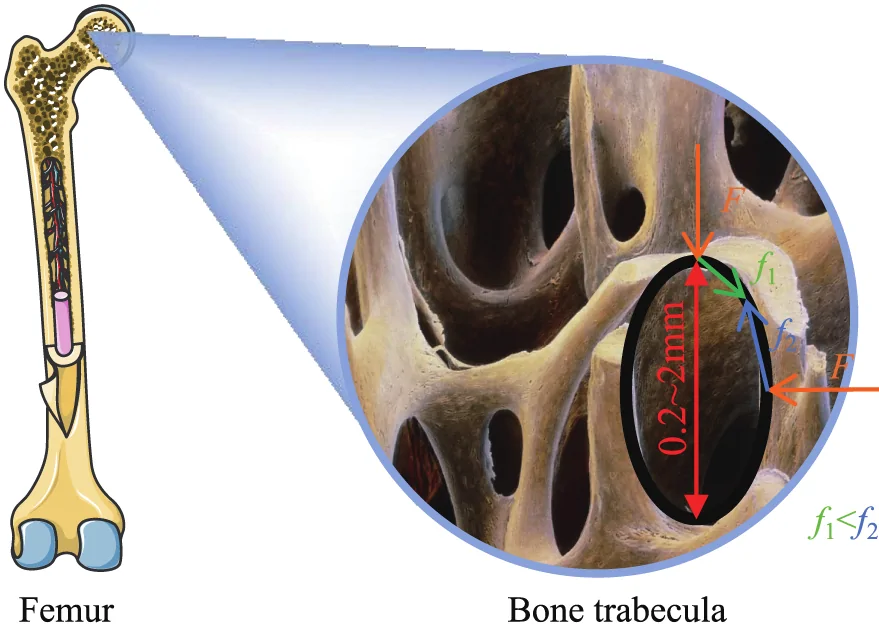
Structure of the cancellous bone of the femur.

Triply periodic minimal surfaces (TPMS) are surfaces with zero mean curvature that are periodic in three mutually orthogonal directions. Each TPMS architecture can be described by a specific implicit function (Equation 1). Porous structures derived from these surfaces possess several advantages, including smooth surfaces, a high specific surface area, and excellent pore connectivity. Unlike traditional strut-based lattices, TPMS structures mitigate stress concentrations at nodal points and more accurately replicate the morphology of native trabecular bone, resulting in a more biomimetic mechanical response. Among the various TPMS architectures, the Gyroid is particularly notable for its mechanical and structural properties that closely resemble those of cancellous bone. Consequently, while Gyroid-based designs have been extensively employed in bone scaffold engineering, their application in fundamental investigations of cancellous bone mechanics remains limited.

$$\phi \left(r\right)=\overset{K}{\underset{k=1}{\sum }}A_{k}\cos\left[2\pi \left(h_{k}\cdot r\right)/\lambda_{k}+p_{k}\right]=C_{\text{P}}.$$ (1)

where r is the location vector in the Euclidean space, $h_{k}$ is the k -th lattice vector in the reciprocal space, $h_{k}\cdot r$ denotes the Euclidean inner product between the *k*th reciprocal-lattice vector, and the spatial position vector $A_{k}$ is the magnitude factor, $\lambda_{k}$ is the periodic wavelength of periods, $p_{k}$ is the phase shift, and $C_{\text{P}}$ is a constant. Thus, specific periodic structures can be constructed based on this implicit form. The implicit function employed for the Gyroid-type TPMS in this study is:

$$\phi \left(x,y,z\right)=\sin\left(2\pi x\right)\cos\left(2\pi y\right)+\sin\left(2\pi y\right)\cos\left(2\pi z\right)+\sin\left(2\pi z\right)\cos\left(2\pi x\right)=C_{\text{P}}.$$ (2)

A TPMS partitions space into two continuous, non-interpenetrating subspaces, as illustrated in Figure 2a. A solid Gyroid porous structure is created by filling one of these subspaces with material (Figure 2b). In clinical imaging and materials science, the degree of anisotropy in porous materials, such as cancellous bone, is quantified using the mean intercept length (MIL) method. For a TPMS-based model, the degree of anisotropy, which is quantifiable by the MIL method, can be directly controlled by the aspect ratios of the unit cell dimensions: length $\left(l_{x}\right)$ , width $\left(l_{y}\right)$ , and height $\left(l_{z}\right)$ . In this study, cancellous bone is modeled as a transversely isotropic material, assuming uniform anisotropy within each sample. The degree of anisotropy (DA) is therefore defined by the aspect ratio $DA=\frac{l_{z}}{l_{x}}=\frac{l_{z}}{l_{y}}$ . Based on the physiological range of pore sizes in cancellous bone, the lateral dimensions were set to $l_{x}=l_{y}=1.8$ mm. Figure 2c illustrates unit cells with two distinct degrees of anisotropy.

**FIGURE 2:**
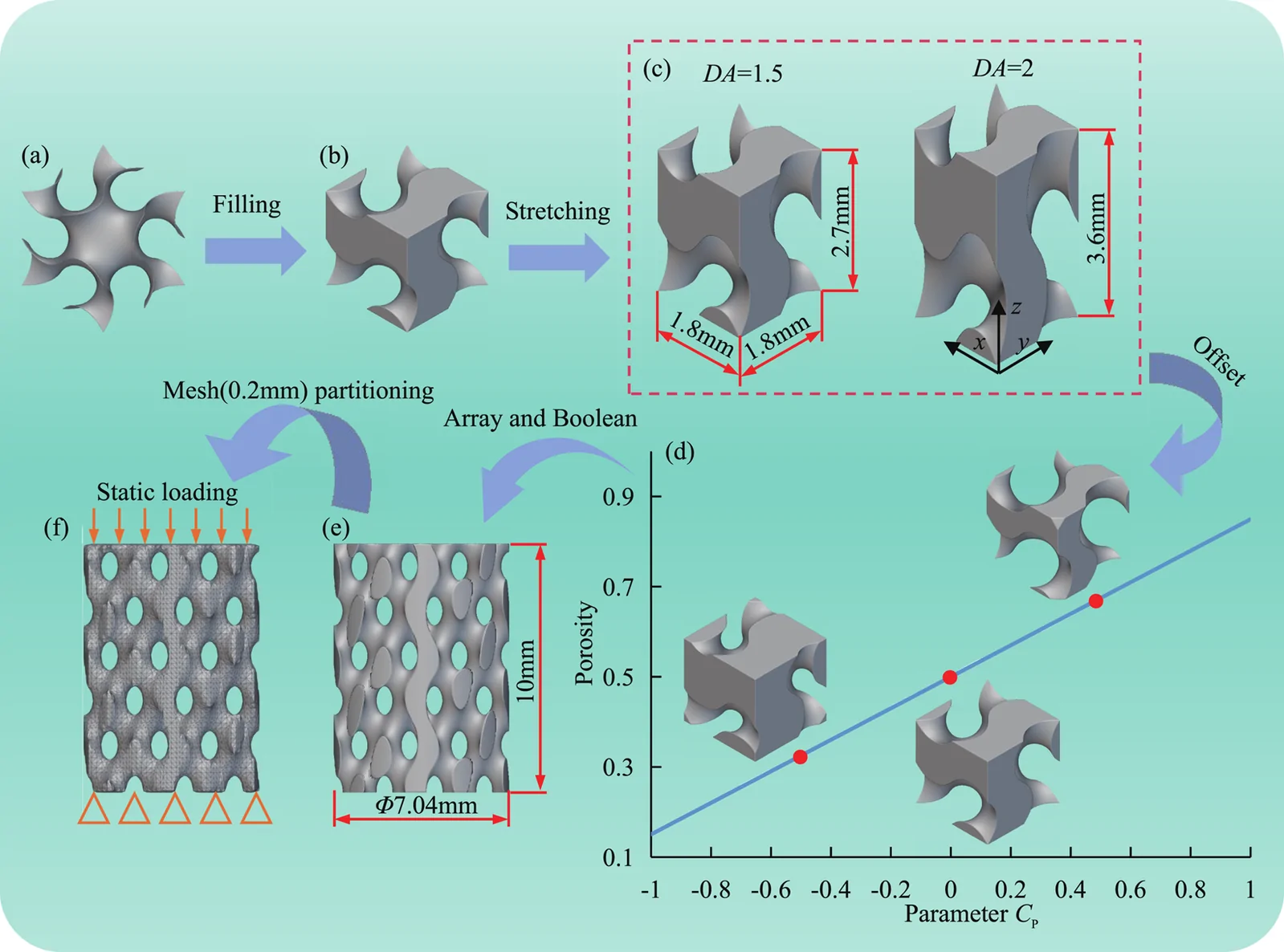
Gyroid porous structure modeling process. **(a)** Gyroid TPMS surface; **(b)** solid Gyroid structure generated by filling one subspace; **(c)** unit cells with *DA* = 1.5 and *DA* = 2.0 generated by stretching; **(d)** relationship between porosity and parameter *C*_P_, with representative offset geometries; **(e)** cylindrical Gyroid model generated by array and Boolean operations and subsequently meshed; **(f)** boundary conditions for static compression.

The porosity of the Gyroid structure is controlled by adjusting the parameter $C_{\text{P}}$ in Equation 2. Modifying $C_{\text{P}}$ applies an offset to the surface, which in turn alters the relative volume of the two subspaces. As depicted in Figure 2d, porosity (P) demonstrates a nearly linear relationship with the parameter $\left(C_{\text{P}}\right)$ , which can be approximated by Equation 3:

$$P=0.3495C_{\text{P}}+0.500.$$ (3)

### 2.2 Finite element modeling

To validate the use of the Gyroid porous structure as a biomimetic model for cancellous bone, finite element simulations were performed to replicate the experimental compression tests reported by (Hambli, 2013). In the referenced study, cylindrical specimens (7.04 mm in diameter, 10 mm in length) were harvested from the proximal femur and the greater trochanter of human cadavers. Uniaxial compression was applied parallel to the principal trabecular orientation. Hambli (2013) reported a mean degree of anisotropy ( $DA_{\text{mean}}$ = 1.8) and three distinct bone volume fractions ( BV/TV = 33.3%, 37.7%, and 39.4%). Accordingly, three corresponding Gyroid porous structures were generated using these parameters, subsequently meshed, and analyzed via static loading simulations (Figures 2d,f).

A mesh sensitivity analysis was performed on the model with BV/TV = 37.7%. The model was meshed with tetrahedral elements at three average sizes (0.15, 0.20, and 0.25 mm), resulting in 229,418, 116,951, and 71,743 elements, respectively. Replicating the experimental boundary conditions, the model’s base was fully constrained, and a uniform axial displacement was applied to its top surface to simulate compression.

The material properties of cancellous bone exhibit significant variability, influenced by factors such as individual differences, anatomical location, age, and health status. Consequently, determining a single, representative value for these properties is challenging. As shown in Table 2, reported values for the Young’s modulus of cancellous bone from the human femur span a broad range, making the selection of a definitive value for finite element simulation problematic. To account for this variability, simulations were conducted on the cancellous bone models using three distinct Young’s modulus values: 15, 20, and 25 GPa. For the material model, the failure strain was set to 1.57% and the Poisson’s ratio to 0.3. Material failure was simulated using an element deletion technique. To replicate the quasi-static compression used in the experimental tests, all simulations were performed using the implicit static solver in LS-DYNA R12.0 (LSTC, Livermore, CA, United States) on a workstation equipped with an Intel Core i5-12600K CPU (3.7 GHz), using 8 CPU cores.

**TABLE 2:** Trabecular Young’s modulus as determined by tests.

Test method	Anatomical site	Gender	Age	Young’s modulus (GPa)	References
Bending	Femur	F	56	10.09 ± 2.42	Carretta et al. (2013)
Tension	Femur	F	56	16.24 ± 2.47	Carretta et al. (2013)
Ultrasonic	Femur	-	-	13.0 ± 1.47	Ashman and Rho (1988)
Ultrasonic	Distal femur	M	65	17.50 ± 1.12	Turner et al. (1999)
Nanoindentation	Femoral neck	Both	53 ∼ 93	11.4 ± 5.6	Zysset et al. (1999)
Nanoindentation	Distal femur	M	65	18.14 ± 1.70	Turner et al. (1999)
Nanoindentation	Femoral neck	Both	27 ∼ 93	11.1 ± 2.4	Hoffler et al. (2000)
Nanoindentation	Proximal femur	Both	73 ∼ 91	19.6 – 22.3	Chevalier et al. (2007)
Nanoindentation	Femoral	F	44, 61, 68	22.34 ± 3.01	Smith et al. (2010)
Nanoindentation	Proximal femur	M	72	16.34 ± 1.76	Jiroušek et al. (2011)
Nanoindentation	Femoral head	M	72	15.39 ± 1.40	Zlámal et al. (2012)
FEM	Femoral neck	Both	51 ∼ 85	18.0 ± 2.8	Bayraktar et al. (2004)

### 2.3 Simulation dataset construction

Previous studies have established that at the mesoscale, the apparent mechanical properties of cancellous bone are governed by four primary parameters: the material-level Young’s modulus $\left(E_{\text{t}}\right)$ and failure strain $\left(\varepsilon_{\text{t}}\right)$ , and the structural parameters of bone volume fraction (BV/TV) and degree of anisotropy (DA) . These inputs determine the apparent properties, principally the apparent Young’s modulus $\left(E_{\text{c}}\right)$ and failure stress $\left(\sigma_{\text{c}}\right)$ . The physiological and structural ranges for these parameters, as reported in the literature, are summarized in Table 3. Using these established ranges, Latin hypercube sampling (LHS) was employed to generate 200 unique sets of input parameters for the simulation dataset.

**TABLE 3:** The range of values for simulation parameters.

Parameter	Range in the literature	References
$E_{\text{t}}$ (GPa)	8–23	Wu et al., (2018)
$\varepsilon_{\text{t}}$ (%)	0.81–2.5	Bevill et al. (2006), Wolfram et al. (2011)
BV/TV	0.16–0.6	Green et al. (2011), Perilli et al. (2008), Wu et al. (2013), Greenwood et al. (2015), Hambli (2013)
DA	1.4–3	Green et al. (2011), Greenwood et al. (2015)

Figure 3 shows a flowchart illustrating the process of generating the dataset and training the model. To facilitate the extraction of apparent mechanical properties, the cancellous bone was modeled as 10 mm × 10 mm × 10 mm cubes. A corresponding parametric Gyroid model was generated and simulated for each parameter combination. The resulting force-displacement data from each simulation were converted into stress-strain curves, from which the apparent Young’s modulus $\left(E_{\text{c}}\right)$ and failure stress $\left(\sigma_{\text{c}}\right)$ were calculated. The engineering stress and engineering strain were calculated as $\sigma =\frac{F}{A_{0}}$ and $\varepsilon =\frac{\delta }{L_{0}}$ , respectively, where F is the axial load, $A_{0}$ is the initial cross-sectional area of the specimen, and $L_{0}$ is its initial height. To minimize the effects of initial contact and nonlinear deformation near the peak stress, data points corresponding to 10% ∼ 60% of the peak stress on the ascending branch of the stress-strain curve were selected. A linear regression was then performed using the least-squares method, as expressed in Equation 4:

$$\sigma_{i}=E_{\text{c}}\varepsilon_{i}+b,$$ (4)

where b is the regression intercept. The elastic modulus was determined from the slope of the fitted line according to Equation 5:

$$E_{\text{c}}=\frac{\overset{n}{\underset{i=1}{\sum }}\left(\varepsilon_{i}-\bar{\varepsilon }\right)\left(\sigma_{i}-\bar{\sigma }\right)}{\overset{n}{\underset{i=1}{\sum }}{\left(\varepsilon_{i}-\bar{\varepsilon }\right)}^{2}},$$ (5)

where n is the number of data points included in the regression, while $\bar{\varepsilon }$ and $\bar{\sigma }$ are the mean strain and mean stress within the selected fitting interval, respectively. The coefficient of determination $R^{2}$ was also calculated to evaluate the quality of the linear fit.

**FIGURE 3:**
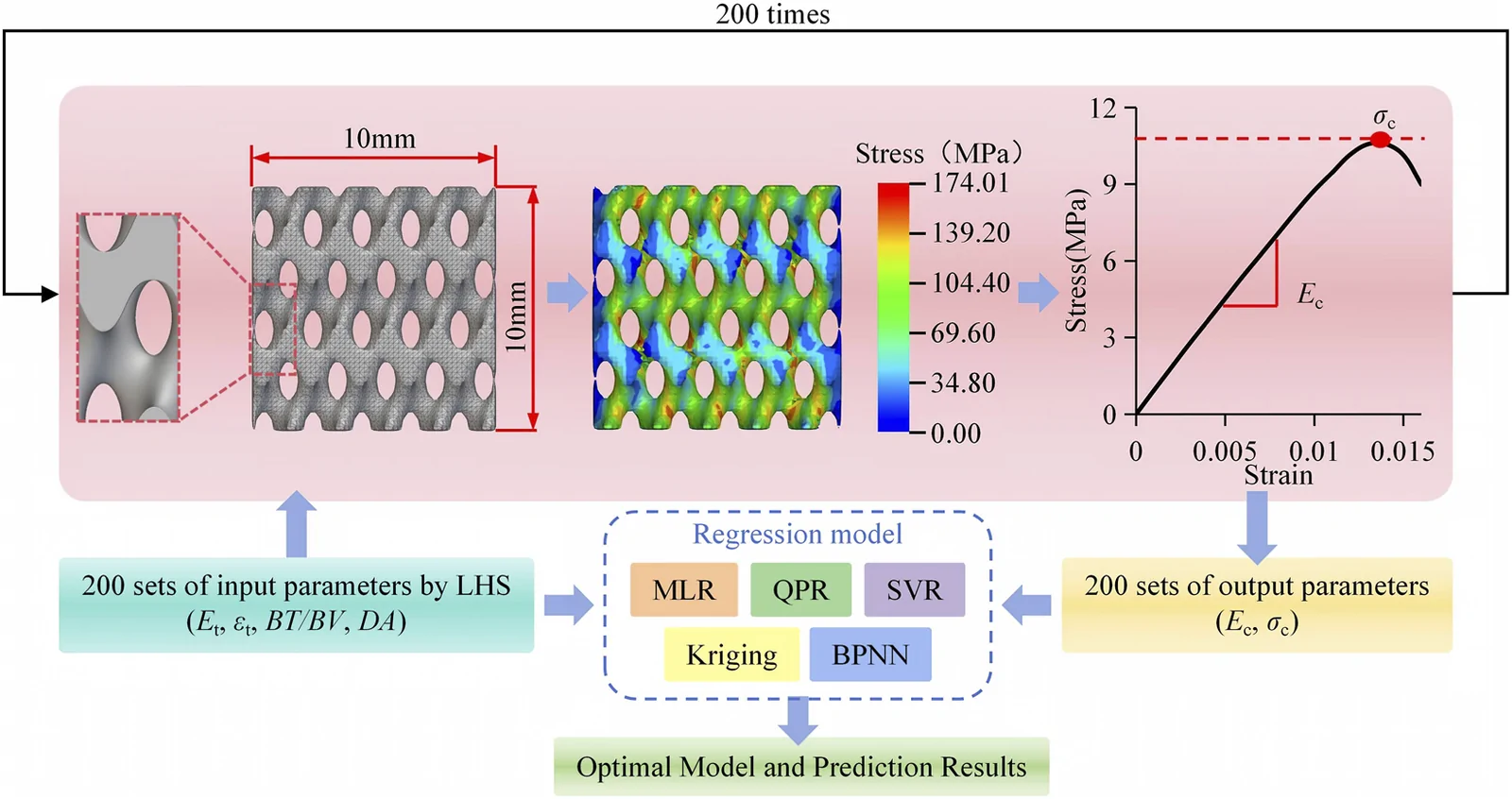
Schematic of the predictive model construction process.

The failure stress of each specimen was defined as the maximum engineering stress recorded during the entire loading process, as given in Equation 6:

$$\sigma_{\text{c}}=\underset{1\leq i\leq N}{\max}\left(\sigma_{i}\right)=\underset{1\leq i\leq N}{\max}\left(\frac{F_{i}}{A_{0}}\right),$$ (6)

where N is the total number of valid data points for the specimen.

### 2.4 Regression model development

Based on the 200 finite-element simulation cases, five regression models were developed to approximate the relationships between the mesoscale parameters and the apparent mechanical responses of cancellous bone. The input vector was defined in Equation 7 as follows:

$$x={\left[E_{\text{t}},\varepsilon_{\text{t}},BV/TV,DA\right]}^{\text{T}}.$$ (7)

The target response y was either the FEA-derived apparent Young’s modulus $E_{\text{c}}$ or failure stress $\sigma_{\text{c}}$ .

The evaluated algorithms included multiple linear regression (MLR), quadratic polynomial regression (QPR), support vector regression (SVR), a Kriging surrogate model, and a backpropagation neural network (BPNN). Separate single-output models were developed for $E_{\text{c}}$ and $\sigma_{\text{c}}$ to permit consistent model optimization and performance comparison. The five models represented increasing levels of flexibility, ranging from explicit linear and quadratic functions to kernel-based, probabilistic, and neural-network approaches. Common procedures for data preprocessing, hyperparameter optimization, and nested cross-validation are described in Section 2.5.

#### 2.4.1 Multiple linear regression

Multiple linear regression was used as the lowest-complexity baseline to determine whether the apparent mechanical responses could be represented by a linear combination of the four input parameters. The model was expressed in Equation 8 as follows:

$${\hat{y}}_{i}=\beta_{0}+\beta_{1}E_{\text{t},i}+\beta_{2}\varepsilon_{\text{t},i}+\beta_{3}{\left(BV/TV\right)}_{i}+\beta_{4}{DA}_{i},$$ (8)

where ${\hat{y}}_{i}$ denotes the predicted value of $E_{\text{c}}$ or $\sigma_{\text{c}}$ for the i th sample, $\beta_{0}$ is the intercept, and $\beta_{1}∼\beta_{4}$ are the regression coefficients corresponding to the four input variables.

For a training set containing n samples, the coefficients were estimated in Equation 8 using ordinary least squares:

$$\hat{\beta }=\arg\underset{\beta }{\min}\overset{n}{\underset{i=1}{\sum }}{\left(y_{i}-{\hat{y}}_{i}\right)}^{2},$$ (9)

where $y_{i}$ is the finite-element-derived response of the *i*th sample, and ${\hat{y}}_{i}$ is the corresponding regression prediction.

#### 2.4.2 Quadratic polynomial regression

Quadratic polynomial regression was employed to account for response curvature and pairwise interactions among the material and structural parameters while retaining an explicit mathematical form. The model was defined in Equation 10 as follows:

$$\hat{y}=\beta_{0}+\overset{4}{\underset{j=1}{\sum }}\beta_{j}x_{j}+\overset{4}{\underset{j=1}{\sum }}\beta_{jj}x_{j}^{2}+\overset{3}{\underset{j=1}{\sum }}\overset{4}{\underset{k=j+1}{\sum }}\beta_{jk}x_{j}x_{k},$$ (10)

where $\beta_{0}$ is the intercept, $\beta_{j}$ represents the coefficient of the *j*th linear term, $\beta_{jj}$ represents the coefficient of the corresponding quadratic term, and $\beta_{jk}$ represents the interaction coefficient between the *j*th and *k*th input variables. The model contained four linear terms, four squared terms, six pairwise interaction terms, and one intercept, resulting in 15 regression coefficients.

The four original inputs were standardized before constructing the polynomial terms to reduce differences in numerical scale and improve the conditioning of the design matrix. The coefficients were subsequently determined using ordinary least squares (Equation 9).

#### 2.4.3 Support vector regression

Support vector regression was used to describe nonlinear input-output relationships by mapping the input variables into a higher-dimensional feature space. For a training set ${\left\{\left(x_{i},y_{i}\right)\right\}}_{i=1}^{n}$ , the η -insensitive SVR formulation was expressed in Equation 11 as follows:

$$\begin{array}{cc} \underset{w,b,\xi_{i},\xi_{i}^{*}}{\min} & \frac{1}{2}{\left\|w\right\|}^{2}+C\overset{n}{\underset{i=1}{\sum }}\left(\xi_{i}+\xi_{i}^{*}\right),\\ subject\;to\; & y_{i}-w^{\text{T}}\phi \left(x_{i}\right)-b\leq \eta +\xi_{i},\\ & w^{\text{T}}\phi \left(x_{i}\right)+b-y_{i}\leq \eta +\xi_{i}^{*},\\ & \xi_{i},\xi_{i}^{*}\geq 0, \end{array}$$ (11)

where ϕ(x) denotes the nonlinear feature mapping, C is the penalty parameter, η defines the width of the error-insensitive region, and $\xi_{i}$ and $\xi_{i}^{*}$ are slack variables for deviations outside this region.

The adopted Gaussian radial basis function kernel is given in Equation 12:

$$K\left(x_{i},x_{j}\right)=\exp\left[-\frac{{\left\|x_{i}-x_{j}\right\|}^{2}}{2\sigma_{k}^{2}}\right],$$ (12)

where $\sigma_{k}$ is the kernel scale. The corresponding prediction function is given in Equation 13:

$$\hat{y}\left(x\right)=\overset{n}{\underset{i=1}{\sum }}\left(\alpha_{i}-\alpha_{i}^{*}\right)K\left(x_{i},x\right)+b,$$ (13)

where $\alpha_{i}$ and $\alpha_{i}^{*}$ are the Lagrange multipliers. Samples with nonzero values of $\alpha_{i}-\alpha_{i}^{*}$ constitute the support vectors and determine the regression function. The penalty parameter C , kernel scale $\sigma_{k}$ , and insensitive-region width η were treated as hyperparameters and optimized through the inner cross-validation procedure described in Section 2.5.

#### 2.4.4 Kriging surrogate model

A Kriging surrogate model was constructed to approximate the deterministic finite-element responses. The response at an input vector x was represented in Equation 14 as follows:

$$y\left(x\right)=\mu +Z\left(x\right),$$ (14)

where μ is an unknown constant trend and Z(x) is a zero-mean Gaussian random process. The covariance between two responses was defined in Equation 15 as follows:

$$Cov\left[Z\left(x_{i}\right),Z\left(x_{j}\right)\right]=\sigma_{Z}^{2}R\left(x_{i},x_{j}\right),$$ (15)

where $\sigma_{Z}^{2}$ is the process variance and R is the correlation function. The adopted anisotropic Gaussian correlation function is given in Equation 16:

$$R\left(x_{i},x_{j}\right)=\exp\left[-\overset{4}{\underset{k=1}{\sum }}\theta_{k}{\left(x_{ik}-x_{jk}\right)}^{2}\right],$$ (16)

where $\theta_{k}$ is the correlation parameter associated with the k th input variable.

For a new input $x_{0}$ , the Kriging prediction was calculated using Equation 17:

$$\hat{y}\left(x_{0}\right)=\hat{\mu }+r^{\text{T}}R^{-1}\left(y-1\hat{\mu }\right),$$ (17)

where R is the correlation matrix of the training samples and r contains the correlations between $x_{0}$ and the training inputs. The constant trend and Gaussian correlation function were specified in advance, while the correlation parameters and process variance were estimated by maximum likelihood using the samples in each training fold. Because the responses were obtained from deterministic finite-element simulations, the nugget term was fixed at a small value solely to improve numerical stability.

#### 2.4.5 Backpropagation neural network

A single-hidden-layer backpropagation neural network was employed to establish a flexible nonlinear mapping between the four input parameters and each mechanical response. Separate networks were constructed for $E_{\text{c}}$ and $\sigma_{\text{c}}$ , each with four input neurons, $n_{\text{h}}$ hidden neurons, and one output neuron.

For the j th hidden neuron, the hidden-layer output was calculated using Equation 18:

$$h_{j}=g\left(\overset{4}{\underset{k=1}{\sum }}w_{jk}x_{k}+b_{j}\right),$$ (18)

and The network prediction was then expressed in Equation 19 as follows:

$$\hat{y}=\overset{n_{\text{h}}}{\underset{j=1}{\sum }}v_{j}h_{j}+b_{o},$$ (19)

where $w_{jk}$ and $v_{j}$ are the input-to-hidden and hidden-to-output weights, respectively; $b_{j}$ and $b_{o}$ are bias terms; and g(⋅) is the hidden-layer activation function. A hyperbolic tangent sigmoid function was used in the hidden layer, and a linear activation function was used in the output layer.

The network parameters were determined by minimizing the mean squared error defined in Equation 20:

$$MSE=\frac{1}{n}\overset{n}{\underset{i=1}{\sum }}{\left(y_{i}-{\hat{y}}_{i}\right)}^{2}.$$ (20)

The Levenberg-Marquardt backpropagation algorithm was used for network training. The number of hidden neurons $n_{\text{h}}$ was treated as the principal structural hyperparameter and was selected through inner cross-validation. Because BPNN training depends on the initial weights and biases, each candidate architecture was trained repeatedly using predetermined random seeds, and its mean validation performance was used for model selection. The repeated-training and stopping procedures are detailed in Section 2.5.

### 2.5 Model training, hyperparameter optimization, and validation

All MATLAB implementations used for model construction, hyperparameter optimization, prediction, and performance evaluation are provided in the Supplementary Material. To ensure a fair comparison, the five regression models were evaluated using identical outer cross-validation partitions and performance metrics. Separate modeling and validation procedures were conducted for the apparent Young’s modulus $E_{\text{c}}$ and failure stress $\sigma_{\text{c}}$ . Multiple linear regression, quadratic polynomial regression, and Kriging were fitted without cross-validation-based hyperparameter selection, whereas the hyperparameters of support vector regression and BPNN were optimized within the inner cross-validation loop. Figure 4 shows the workflow for regression-model development and comparison.

**FIGURE 4:**
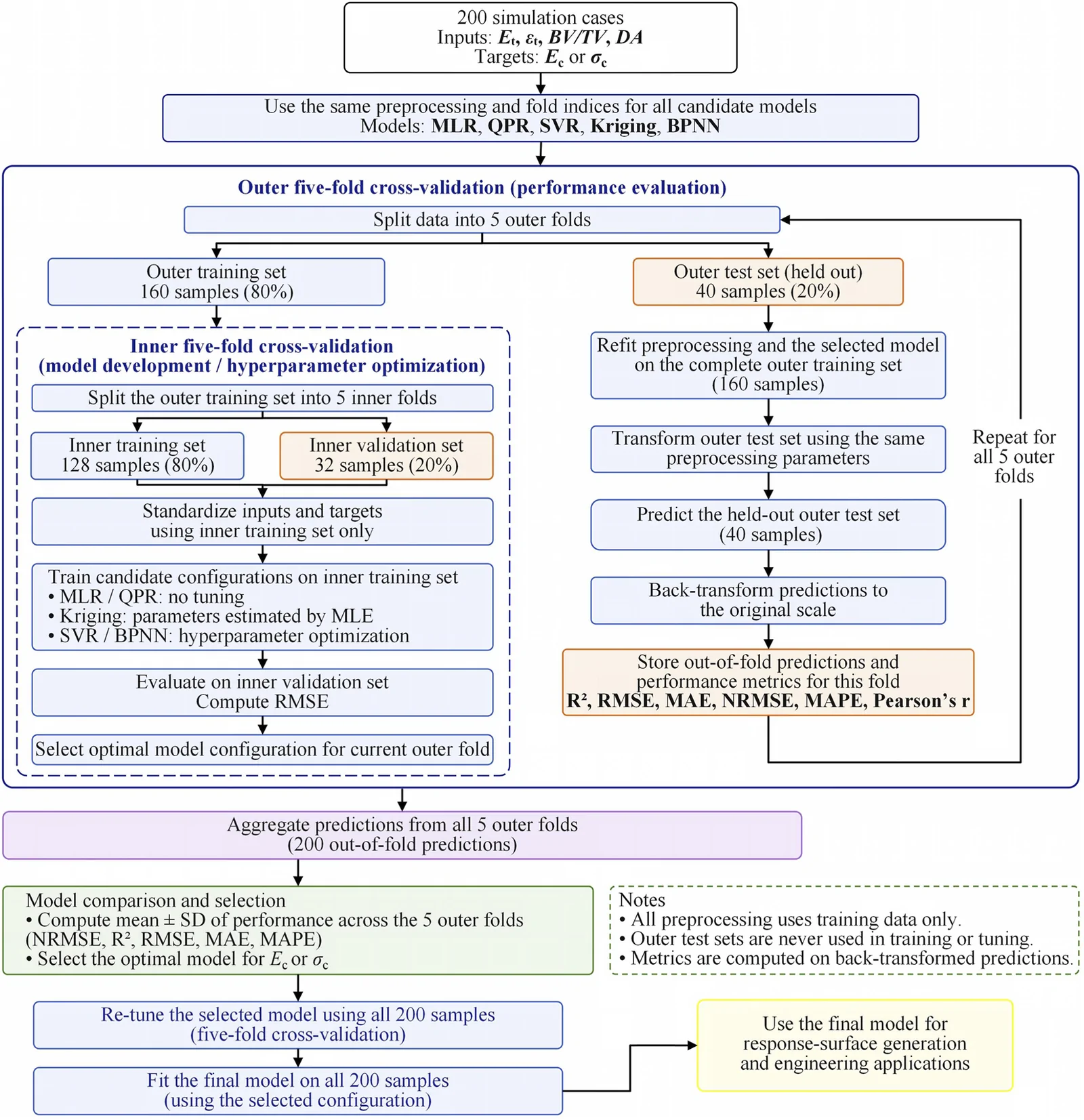
Nested five‐fold cross-validation workflow for regression-model development and comparison.

#### 2.5.1 Data preprocessing

The four input variables and each target response were standardized within each training fold using the Z-score transformation given in Equation 21:

$$\left\{\begin{array}{c} x_{ij}^{*}=\frac{x_{ij}-\mu_{x_{j}}}{s_{x_{j}}}\\ y_{i}^{*}=\frac{y_{i}-\mu_{y}}{s_{y}}, \end{array}\right.$$ (21)

where $x_{ij}$ is the j th input variable of the *i*th sample, and $\mu_{x_{j}}$ and $s_{x_{j}}$ are the mean and standard deviation of the j th input calculated exclusively from the corresponding training fold. Similarly, $\mu_{y}$ and $s_{y}$ are the mean and standard deviation of the target response in that training fold.

The transformation parameters obtained from the training samples were applied unchanged to the associated validation or test samples. Predicted responses were subsequently transformed back to their original units. For the quadratic polynomial regression model, the linear, squared, and interaction terms were generated after standardizing the four original input variables. No information from the validation or outer test samples was used to calculate the preprocessing parameters.

#### 2.5.2 Nested five-fold cross-validation

Nested five-fold cross-validation was adopted to separate hyperparameter selection from model performance evaluation. The complete dataset of 200 simulation cases was first divided into five mutually exclusive outer folds, each containing 40 samples. In each outer iteration, four folds containing 160 samples were used for model development, and the remaining 40 samples formed the outer test set. The procedure was repeated until each sample had been used once for outer testing.

For models requiring hyperparameter optimization, the 160 outer-training samples were further divided into five inner folds. In each inner iteration, 128 samples were used for model fitting and 32 samples were used for validation. Candidate hyperparameter configurations were evaluated across all five inner folds, and the configuration with the lowest mean validation error was selected. The selected configuration was then refitted using the complete outer-training set and evaluated on the held-out outer test set.

The same outer-fold indices were used for all five algorithms and both target responses. The same inner-fold indices were also used when optimizing SVR and BPNN, thereby ensuring that differences in predictive performance were attributable to the regression algorithms rather than to variations in data partitioning. A fixed random seed was used to generate and preserve all cross-validation partitions.

#### 2.5.3 Hyperparameter optimization

The inner-loop objective was the mean normalized root-mean-square error (NRMSE), as defined in Equation 22:

$$NRMSE=\frac{RMSE}{s_{y,train}},$$ (22)

where $s_{y,train}$ is the standard deviation of the target response calculated from the corresponding inner-training fold. For a candidate hyperparameter configuration θ , its inner cross-validation score was calculated using Equation 23:

$$J\left(\theta \right)=\frac{1}{5}\overset{5}{\underset{q=1}{\sum }}{NRMSE}_{q}\left(\theta \right),$$ (23)

where q denotes the inner fold. The configuration minimizing J(θ) was selected.

For SVR, the penalty parameter C , Gaussian kernel scale $\sigma_{k}$ , and insensitive-region width η were optimized within the ranges specified in Equation 24:

$$C\in \left[10^{-2},10^{3}\right],\;\sigma_{k}\in \left[10^{-2},10^{2}\right],\;\eta \in \left[10^{-3},10^{-1}\right].$$ (24)

The three parameters were searched on logarithmic scales using Bayesian optimization, with the mean inner-fold NRMSE as the objective function.

For BPNN, the number of hidden neurons was selected from the range specified in Equation 25:

$$n_{\text{h}}\in \left\{3,4,\ldots ,15\right\}.$$ (25)

The hidden-layer and output-layer activation functions and the Levenberg-Marquardt training algorithm were fixed as described in Section 2.4.5. Each candidate architecture was trained ten times using predetermined random seeds, and the mean validation NRMSE across the repeated initializations and five inner folds was used for architecture selection.

#### 2.5.4 BPNN training and stopping criteria

Because BPNN performance depends on the initial weights and biases, repeated training was incorporated into both hyperparameter selection and outer-fold evaluation. Within each training dataset, a fixed subset of the available training samples was used for early-stopping validation, while the inner validation or outer test samples remained completely excluded from network fitting.

Network training was terminated when one of the following criteria was reached:

1. The maximum number of training epochs was reached;
2. The gradient fell below the prescribed minimum threshold;
3. The validation loss failed to improve for a predefined number of consecutive iterations.

For each selected architecture, ten independently initialized networks were trained using fixed random seeds. Their predictions were averaged to obtain the BPNN response for the corresponding validation or outer test samples. This procedure reduced the sensitivity of the reported results to a favorable or unfavorable single initialization.

#### 2.5.5 Outer-fold prediction and performance evaluation

After model-specific fitting or inner-loop optimization, each model was reconstructed using the complete outer-training set and then applied to the corresponding outer test set. The resulting predictions were converted back to the original physical units and retained as out-of-fold predictions. After completing all five outer folds, every sample had one prediction generated by a model that had not used that sample during fitting or hyperparameter selection.

Predictive performance was evaluated separately for $E_{\text{c}}$ and $\sigma_{\text{c}}$ using the coefficient of determination $R^{2}$ , root-mean-square error (RMSE), mean absolute error (MAE), NRMSE, mean absolute percentage error (MAPE), and Pearson correlation coefficient r . The metrics were calculated were calculated as shown in Equation 26:

$$\left\{\begin{array}{c} R^{2}=1-\frac{\sum_{i=1}^{m}{\left(y_{i}-{\hat{y}}_{i}\right)}^{2}}{\sum_{i=1}^{m}{\left(y_{i}-\bar{y}\right)}^{2}}\\ RMSE=\sqrt{\frac{1}{m}\overset{m}{\underset{i=1}{\sum }}{\left(y_{i}-{\hat{y}}_{i}\right)}^{2}}\\ MAE=\frac{1}{m}\overset{m}{\underset{i=1}{\sum }}|y_{i}-{\hat{y}}_{i}|\\ MAPE=\frac{100}{m}\overset{m}{\underset{i=1}{\sum }}\left|\frac{y_{i}-{\hat{y}}_{i}}{y_{i}}\right|, \end{array}\right.$$ (26)

where m is the number of samples in the current outer test fold, $y_{i}$ is the FEA-derived response, ${\hat{y}}_{i}$ is the predicted response, and $\bar{y}$ is the mean FEA-derived response in that fold.

The metrics from the five outer folds were summarized as the mean and standard deviation. In addition, the complete set of 200 out-of-fold predictions was used to generate predicted-versus-FEA scatter plots and residual distributions. Adjusted $R^{2}$ was not used for model comparison because the effective number of parameters is not defined consistently across the nonlinear algorithms.

#### 2.5.6 Model selection and final model construction

The optimal model was selected separately for $E_{\text{c}}$ and $\sigma_{\text{c}}$ . Mean outer-fold NRMSE was used as the primary criterion, while $R^{2}$ , RMSE, MAPE, fold-to-fold variability, and model complexity were considered as secondary criteria. Consequently, the two mechanical responses were not required to share the same optimal regression algorithm.

After the optimal algorithm had been identified for each response, its final configuration was determined using all 200 samples. For SVR and BPNN, an additional ordinary five-fold cross-validation was performed on the complete dataset to select the final hyperparameters. MLR and QPR were fitted directly using all samples, and the parameters of the fixed Kriging formulation were re-estimated by maximum likelihood using the complete dataset. The final models were then trained using all 200 simulation cases and retained for subsequent response-surface analysis.

Performance obtained during this final full-dataset fitting stage was not used as evidence of generalization capability. The predictive performance reported in the Results section was based exclusively on the outer test predictions generated by nested cross-validation.

## 3 Results and discussion

### 3.1 Verification of the finite-element model

As shown in Figure 5, the three meshes produced closely comparable force-displacement responses, with a maximum difference of less than 4% in the peak compressive reaction force. This limited mesh dependence indicates that further refinement had only a minor influence on the predicted global mechanical response. Accordingly, an average element size of 0.20 mm was adopted for the subsequent finite-element simulations as a compromise between computational accuracy and efficiency.

**FIGURE 5:**
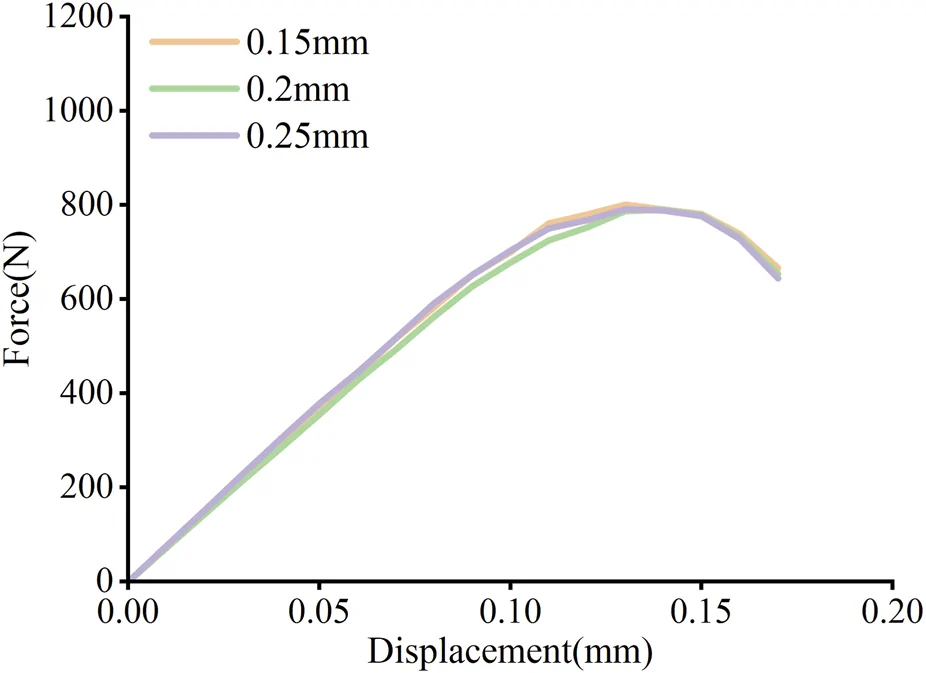
Mesh sensitivity analysis results.

The validation results are presented in Figure 6. The simulation results show strong agreement with the experimental curves, replicating the overall trend and stiffness profile. Despite the broad range of material elastic moduli used, the experimental curves consistently fall near the upper bound of the simulated response envelope, which may reflect relatively high tissue stiffness in the tested specimens or differences in load transfer between the smooth periodic Gyroid architecture and native cancellous bone. Nevertheless, the simulation results effectively bound the experimental data, validating the modeling approach. Overall, the strong correspondence between the simulation and experimental data indicates that the Gyroid porous structure can effectively serve as a surrogate model for predicting the mechanical behavior of cancellous bone.

**FIGURE 6:**
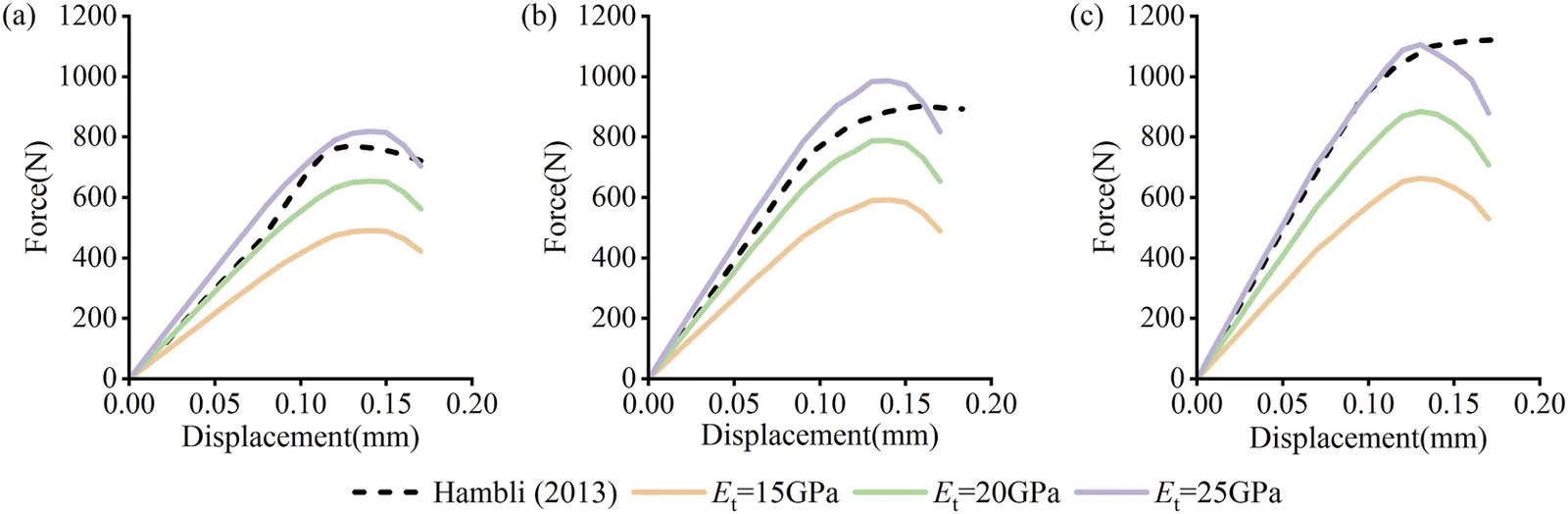
Comparison of simulated and experimental force-displacement responses for **(a)** *BV/TV* = 33.3%, **(b)** *BV/TV* = 37.7%, and **(c)** *BV/TV* = 39.4%, with *DA* = 1.8. The dashed black curves represent the experimental results reported by Hambli (2013), whereas the colored curves represent the simulations with *E*_t_ = 15, 20, and 25 GPa.

### 3.2 Characteristics of the LHS-generated simulation database

A total of 200 input combinations were generated by Latin hypercube sampling within the prescribed ranges of $E_{\text{t}}$ , $\varepsilon_{\text{t}}$ , BV/TV , and DA . As shown in Figure 7, the samples were broadly distributed throughout the four-dimensional parameter space without evident clustering or large unsampled regions. The absolute Pearson correlation coefficients among the four inputs ranged from 0.0049 to 0.087, indicating low pairwise linear collinearity and providing a suitable basis for subsequent multivariable regression analysis.

**FIGURE 7:**
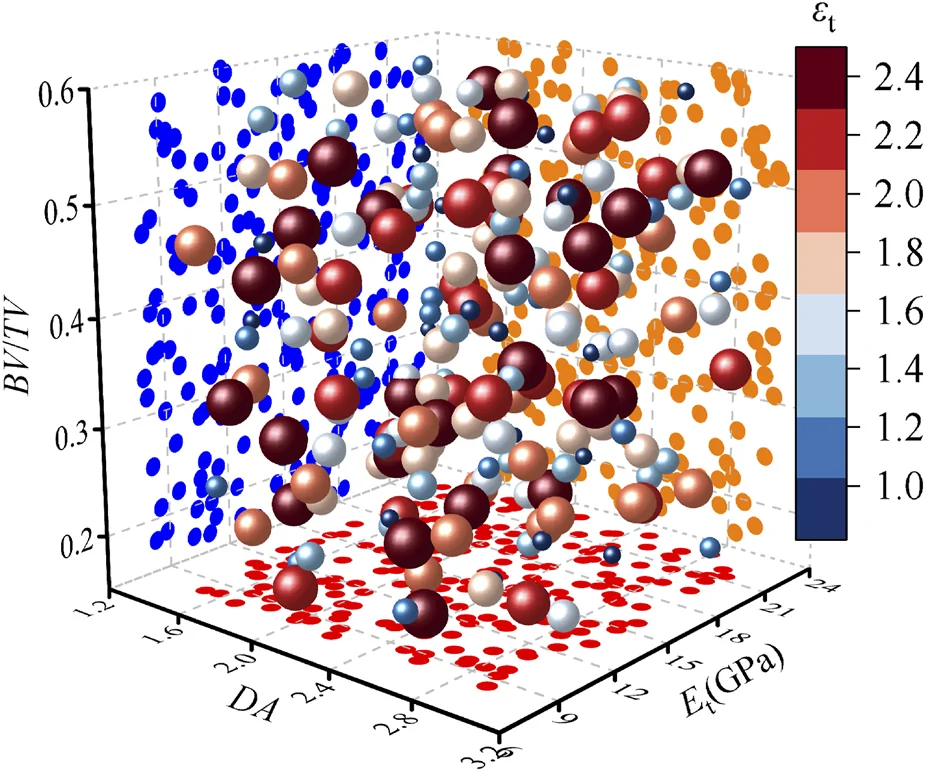
Distribution of the 200 LHS-generated input combinations.

The Pearson correlation matrix in Figure 8 shows distinct associations between the input parameters and the two apparent mechanical responses. BV/TV exhibited the strongest positive correlation with both $E_{\text{c}}$ ( r = 0.85) and $\sigma_{\text{c}}$ ( r = 0.72), suggesting that bone volume fraction was the principal linear correlate of the apparent stiffness and failure stress within the investigated parameter space. $E_{\text{t}}$ was moderately correlated with $E_{\text{c}}$ ( r = 0.49) and $\sigma_{\text{c}}$ ( r = 0.45). By contrast, $\varepsilon_{\text{t}}$ showed almost no linear correlation with $E_{\text{c}}$ ( r = −0.048) but a positive correlation with $\sigma_{\text{c}}$ ( r = 0.36), consistent with its direct relevance to tissue failure rather than the initial elastic response. Relatively weak positive correlations were observed between DA and $E_{\text{c}}$ ( r = 0.24) and between DA and $\sigma_{\text{c}}$ ( r = 0.26).

**FIGURE 8:**
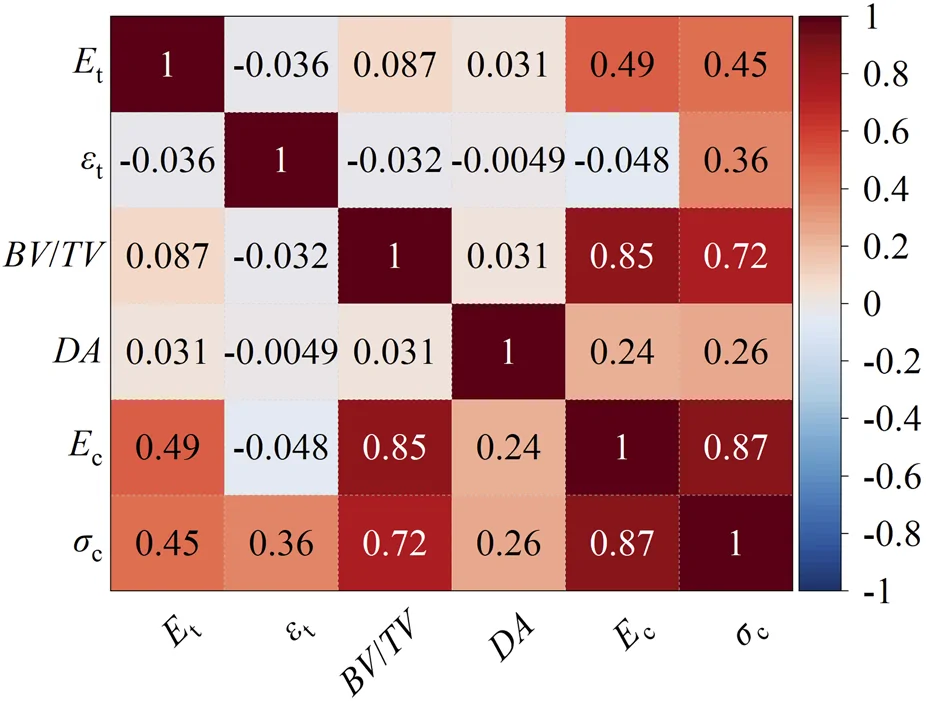
Pearson correlation matrix of the input and output parameters.

A strong positive correlation was also observed between $E_{\text{c}}$ and $\sigma_{\text{c}}$ ( r = 0.87), indicating that samples with greater apparent stiffness generally sustained higher failure stresses. Nevertheless, Pearson coefficients quantify only pairwise linear associations and do not fully capture nonlinear effects or interactions among the four input parameters.

### 3.3 Predictive performance analysis

The predictive performance of MLR, QPR, SVR, Kriging, and BPNN was evaluated separately for $E_{\text{c}}$ and $\sigma_{\text{c}}$ using the outer-test predictions obtained from nested five-fold cross-validation. The metrics in Table 4 are reported as the mean and standard deviation across the five outer folds. Mean NRMSE was used as the primary comparison criterion, with $R^{2}$ , RMSE, MAE, MAPE, Pearson r , and fold-to-fold variability serving as complementary measures of accuracy and stability.

**TABLE 4:** Predictive performance of different models for $E_{\text{c}}$ and $\sigma_{\text{c}}$

Output	Models	$R^{2}$	RMSE (MPa)	MAE (MPa)	NRMSE	MAPE (%)	Pearson r
$E_{\text{c}}$	MLR	0.9303±0.0104	310.90±39.78	250.12±24.24	0.2600±0.0393	28.56±10.03	0.9676±0.0048
	QPR	0.9981±0.0006	50.93±4.59	37.04±3.70	0.0425±0.0037	3.77±0.89	0.9991±0.0003
	SVR	0.9990±0.0004	35.27±5.27	24.82±3.28	0.0294±0.0040	1.73±0.55	0.9996±0.0002
	Kriging	0.9963±0.0026	67.98±25.00	46.80±13.85	0.0568±0.0211	5.21±3.19	0.9984±0.0012
	BPNN	0.9993±0.0003	30.69±3.50	22.66±2.35	0.0256±0.0027	1.68±0.38	0.9997±0.0002
$\sigma_{\text{c}}$	MLR	0.8764±0.0194	5.13±0.39	3.85±0.48	0.3454±0.0282	35.18±13.79	0.9397±0.0081
	QPR	0.9907±0.0030	1.39±0.21	1.01±0.18	0.0937±0.0137	9.20±4.20	0.9959±0.0015
	SVR	0.9987±0.0004	0.52±0.05	0.36±0.03	0.0352±0.0030	2.23±0.45	0.9994±0.0001
	Kriging	0.9926±0.0007	1.26±0.12	0.95±0.12	0.0850±0.0093	6.58±1.17	0.9968±0.0003
	BPNN	0.9978±0.0009	0.67±0.11	0.47±0.07	0.0449±0.0071	2.87±0.79	0.9990±0.0004

For $E_{\text{c}}$ , BPNN achieved the best overall performance, with $R^{2}=0.9993\pm 0.0003$ , RMSE =30.69±3.50 MPa , MAE = 22.66±2.35 MPa , NRMSE =0.0256±0.0027 , and MAPE = 1.68±0.38% . SVR produced comparable results, with an NRMSE of 0.0294±0.0040 and a MAPE of 1.73±0.55% . QPR also provided high predictive accuracy, and Kriging showed a larger mean error and greater fold-to-fold variation, particularly in RMSE and NRMSE. By contrast, MLR performed substantially worse, with an NRMSE of 0.2600±0.0393 and a MAPE of 28.56±10.03% . The marked improvement from MLR to QPR indicates that quadratic effects and pairwise interactions contributed substantially to the relationship between the input parameters and apparent stiffness, while the further gains obtained by SVR and BPNN reflect additional nonlinear behavior.

For $\sigma_{\text{c}}$ , SVR achieved the lowest prediction error, with $R^{2}=0.9987\pm 0.0004$ , RMSE =0.52±0.05 MPa , MAE = 0.36±0.03 MPa , NRMSE =0.0352±0.0030 , and MAPE = 2.23±0.45% . BPNN ranked second, with an NRMSE of 0.0449±0.0071 , followed by Kriging and QPR. MLR again showed the poorest performance, with an NRMSE of 0.3454±0.0282 and a MAPE of 35.18±13.79% . For all five algorithms, the NRMSE for $\sigma_{\text{c}}$ exceeded that for $E_{\text{c}}$ , indicating that failure stress was more difficult to predict than apparent Young’s modulus. This difference is consistent with the stronger dependence of failure behavior on nonlinear parameter interactions and tissue failure strain.

The out-of-fold prediction plots in Figure 9 provide a direct comparison between the finite-element responses and model predictions. Here, $E_{\text{c}}^{\text{FEA}}$ and $\sigma_{\text{c}}^{\text{FEA}}$ denote the finite-element results. $E_{\text{c}}^{\text{OOF}}$ and $\sigma_{\text{c}}^{\text{OOF}}$ denote the corresponding out-of-fold predictions. The predictions from QPR, SVR, Kriging, and BPNN were concentrated near the identity line, while MLR showed clear deviations, particularly at high response values, where it tended to underestimate both $E_{\text{c}}$ and $\sigma_{\text{c}}$ . The close alignment obtained by BPNN for $E_{\text{c}}$ and by SVR for $\sigma_{\text{c}}$ was consistent with their superior error metrics.

**FIGURE 9:**
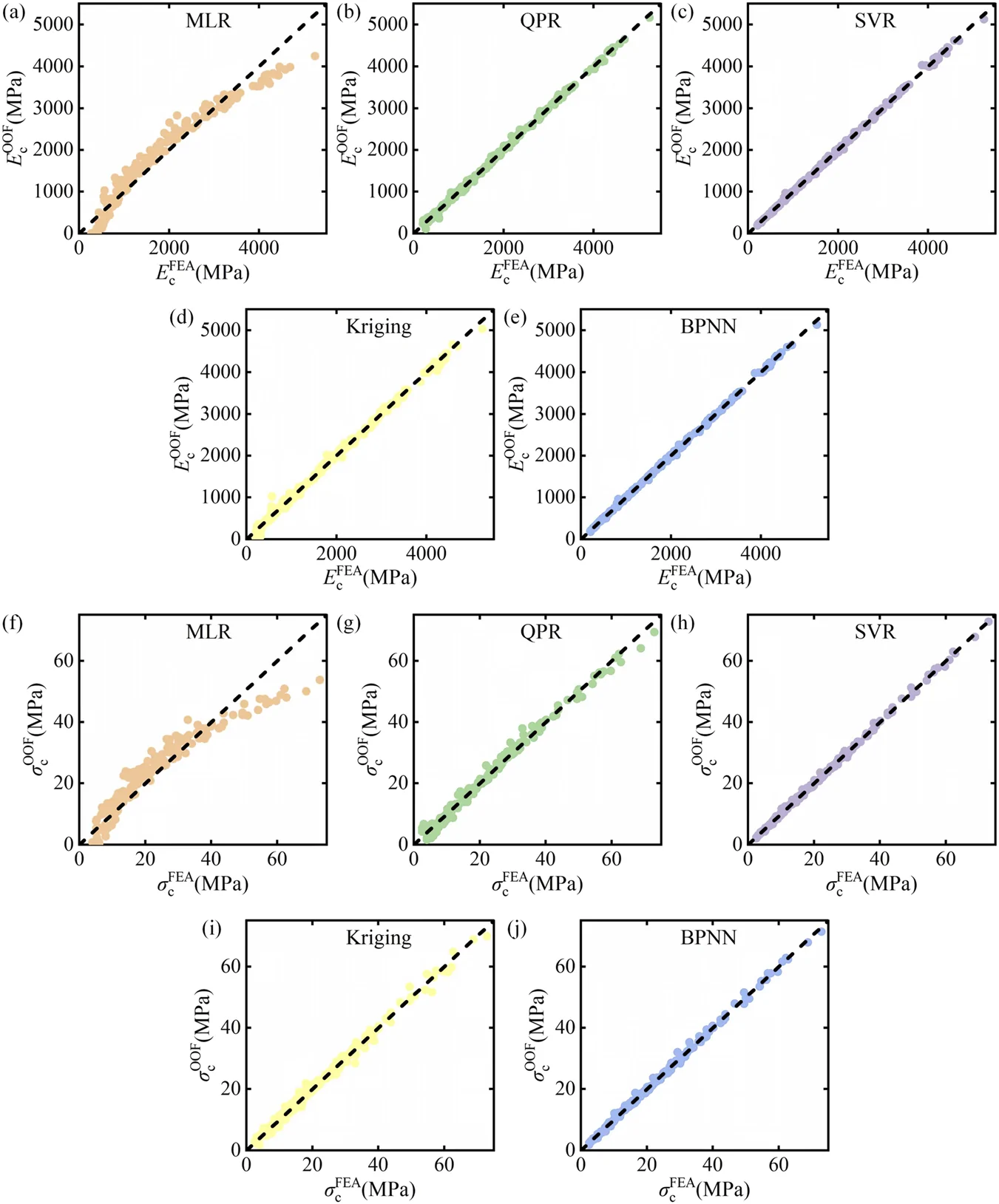
Out-of-fold predictions of the five regression models. **(a–e)** Predictions of *E*_c_ using MLR, QPR, SVR, Kriging, and BPNN, respectively; **(f–j)** predictions of *σ*_c_ using MLR, QPR, SVR, Kriging, and BPNN, respectively. The dashed lines represent the identity line.

The residual distributions in Figure 10 further distinguish the models. The prediction residuals were defined as $e_{E_{\text{c}}}=E_{\text{c}}^{\text{OOF}}-E_{\text{c}}^{\text{FEA}}$ and $e_{\sigma_{\text{c}}}=\sigma_{\text{c}}^{\text{OOF}}-\sigma_{\text{c}}^{\text{FEA}}$ . The quantities $f_{E_{\text{c}}}$ and $f_{\sigma_{\text{c}}}$ represent the probability density functions of the corresponding residuals. MLR exhibited the broadest residual distributions for both outputs, confirming its limited ability to represent the nonlinear response. The residuals of QPR, SVR, Kriging, and BPNN were more concentrated around zero, with BPNN producing the narrowest distribution for $E_{\text{c}}$ and SVR producing the narrowest distribution for $\sigma_{\text{c}}$ . Kriging showed wider tails and greater variability than the leading models, particularly for $E_{\text{c}}$ .

**FIGURE 10:**
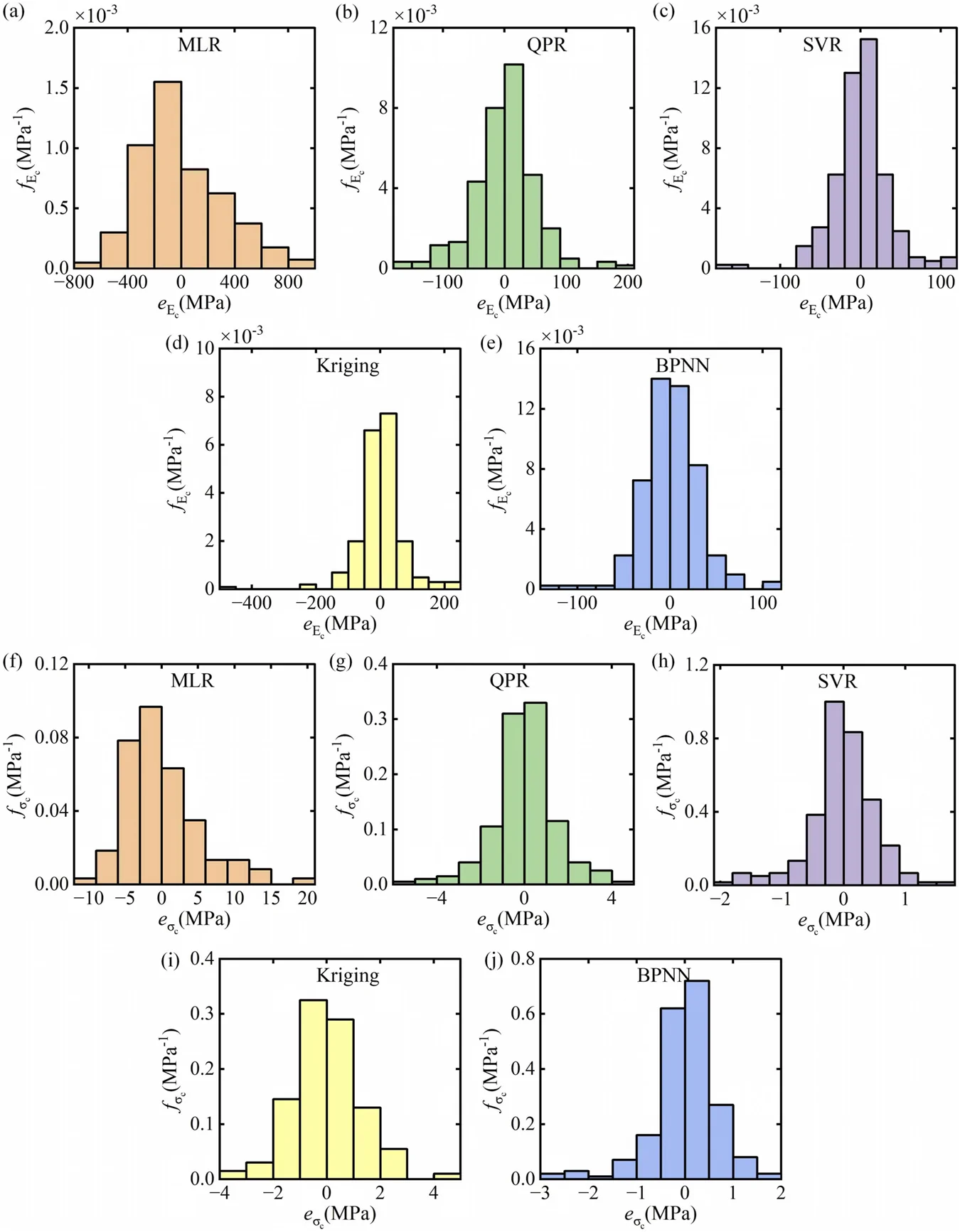
Residual distributions of the out-of-fold predictions. **(a–e)** Residual distributions of *E*_c_ predicted by MLR, QPR, SVR, Kriging, and BPNN, respectively; **(f–j)** residual distributions of *σ*_c_ predicted by MLR, QPR, SVR, Kriging, and BPNN, respectively.

The NRMSE boxplots in Figure 11 confirm these trends. BPNN yielded the lowest median NRMSE and a narrow interquartile range for $E_{\text{c}}$ , and SVR provided the lowest and most stable NRMSE for $\sigma_{\text{c}}$ . Kriging exhibited comparatively large fold-to-fold variability for $E_{\text{c}}$ , while MLR remained clearly separated from the other models for both outputs. Although Pearson r was high for all models, the relatively large errors of MLR demonstrate that correlation alone is insufficient for assessing predictive accuracy.

**FIGURE 11:**
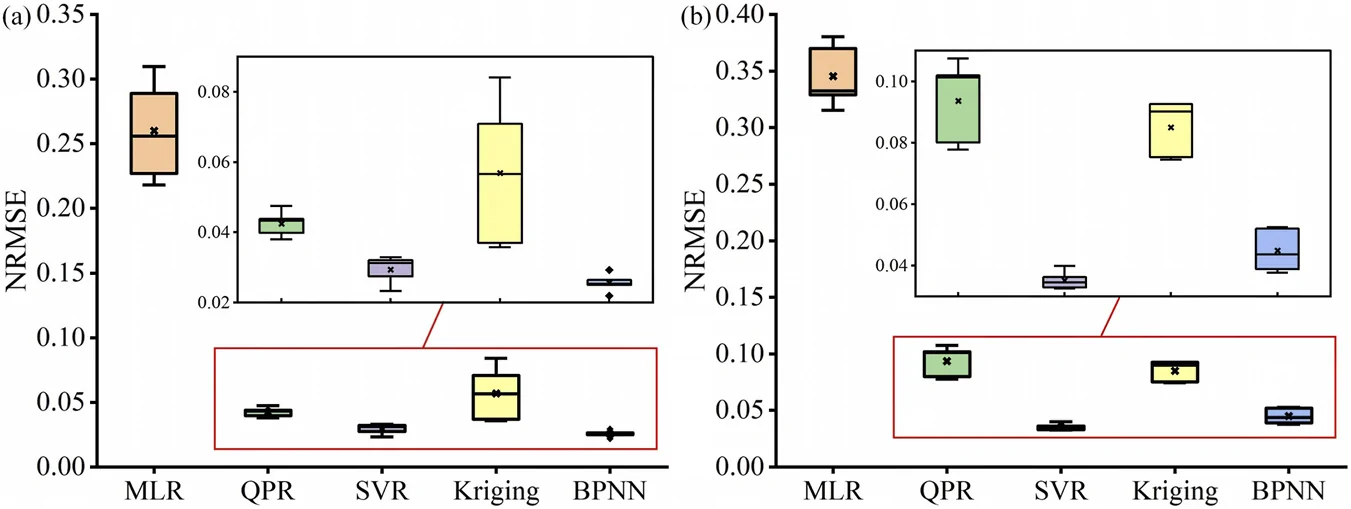
Outer-fold NRMSE distributions of the five regression models **(a)** $E_{\text{c}}$ . **(b)** $\sigma_{\text{c}}$ .

Overall, the results show that the parameter-response relationships cannot be adequately represented by a purely linear model. QPR captured a substantial proportion of the nonlinear behavior while retaining an explicit regression form, and SVR and BPNN provided the highest predictive accuracy. Based on the mean outer-fold NRMSE and the associated stability measures, BPNN for $E_{\text{c}}$ and SVR for $\sigma_{\text{c}}$ were selected for final configuration and response-surface analysis in Section 3.4.

### 3.4 Optimal models and response-surface interpretation

The selection of the optimal model and hyperparameters is summarized in Table 5. The selected models were subsequently retrained using all 200 simulation samples for response-surface analysis.

**TABLE 5:** Optimal models and final configuration.

Target	Selected model	Final configuration	Mean outer NRMSE
$E_{\text{c}}$	BPNN	$n_{\text{h}}=7$	0.025583
$\sigma_{\text{c}}$	SVR	Gaussian kernel; C=995.0896083 $\sigma_{k}=9.7931279$ ; η=0.00106410	0.035240

Response surfaces were generated using the final model selected for each target response. For each analysis, two input variables were varied over their respective sampled ranges using an evenly spaced 50 × 50 grid, while the remaining two variables were fixed at their median sampled values. The resulting input combinations were transformed using the preprocessing parameters of the final model and submitted to the trained surrogate to predict $E_{\text{c}}$ and $\sigma_{\text{c}}$ .

Figures Figures 12a–f show the predicted variation in $E_{\text{c}}$ . Apparent Young’s modulus increased markedly with both $E_{\text{t}}$ and BV/TV , and the curved surface in Figure 12a indicates a positive interaction between tissue stiffness and bone volume fraction. The effect of $E_{\text{t}}$ became more pronounced at higher BV/TV , where a greater proportion of solid material contributed to load transfer. Increasing DA also increased $E_{\text{c}}$ , although its effect was weaker than those of $E_{\text{t}}$ and BV/TV . By contrast, variation in $\varepsilon_{\text{t}}$ produced only minor changes in $E_{\text{c}}$ , as shown in Figures Figures 12c–e. These trends are consistent with the correlation analysis in Section 3.2, in which BV/TV and $E_{\text{t}}$ showed the strongest associations with $E_{\text{c}}$ , whereas $\varepsilon_{\text{t}}$ exhibited little linear correlation.

**FIGURE 12:**
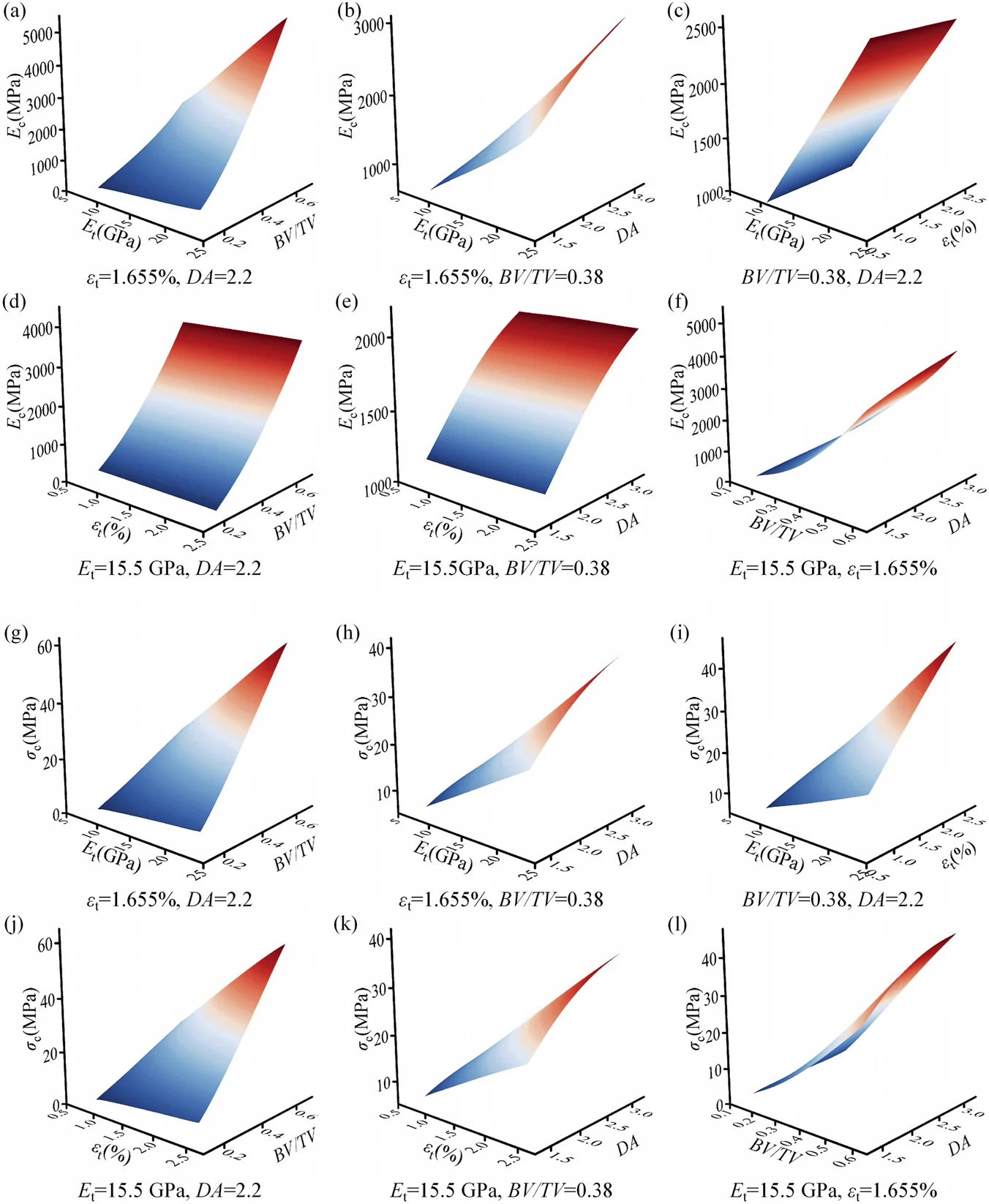
Predicted response surfaces for apparent Young’s modulus and failure stress. **(a–f)** Predicted *E*_c_ as functions of **(a)** *E*_t_ and *BV/TV*, **(b)** *E*_t_ and *DA*, **(c)** *E*_t_ and *ε*_t_, **(d)** *ε*_t_ and *BV/TV*, **(e)** *ε*_t_ and *DA*, and **(f)** *BV/TV* and *DA*. **(g–l)** Predicted *σ*_c_ for the corresponding parameter pairs: **(g)** *E*_t_ and *BV/TV*, **(h)** *E*_t_ and *DA*, **(i)** *E*_t_ and *ε*_t_, **(j)** *ε*_t_ and *BV/TV*, **(k)** *ε*_t_ and *DA*, and **(l)** *BV/TV* and *DA*. The two remaining parameters in each panel were fixed at their median sampled values, as indicated below each subplot.

Figures Figures 12g–l present the corresponding surfaces for $\sigma_{\text{c}}$ . Failure stress increased with $E_{\text{t}}$ , $\varepsilon_{\text{t}}$ , BV/TV , and DA , although the magnitudes of their effects differed. The strongest increases were associated with BV/TV and $E_{\text{t}}$ , reflecting the combined contributions of load-bearing material content and tissue-level stiffness. Unlike $E_{\text{c}}$ , $\sigma_{\text{c}}$ was also sensitive to $\varepsilon_{\text{t}}$ . In particular, the curved $E_{\text{t}}$ - $\varepsilon_{\text{t}}$ surface indicates that the increase in failure stress associated with tissue failure strain became greater as tissue stiffness increased. This behavior is consistent with the dependence of the tissue stress scale on both elastic modulus and strain before failure.

The BV/TV - DA surfaces for both responses exhibited positive gradients, indicating that structural anisotropy modified the mechanical contribution of bone volume fraction in the loading direction. Nevertheless, the influence of DA remained smaller than that of BV/TV , suggesting that the amount of load-bearing material was the dominant structural factor within the investigated parameter range. Overall, the response surfaces demonstrate that the apparent stiffness and failure stress of the Gyroid models were governed by coupled tissue-level and structural parameters. $E_{\text{t}}$ and BV/TV primarily controlled the elastic response, whereas $\varepsilon_{\text{t}}$ contributed more directly to failure stress. Collectively, these findings suggest that variations in mesoscopic parameters, independent of bone mineral density, can significantly influence mechanical performance, which may help to explain why fracture risk varies among individuals or anatomical sites with comparable bone mineral density measurements.

### 3.5 Computational efficiency and potential for rapid prediction

The computational costs of the finite-element workflow are summarized in Table 6. For a single parameter combination, three-dimensional geometry generation and finite-element model preparation required 15.40 and 10.20 min, respectively. The mean solution time of the 200 finite-element models was 13.76 min per case.

**TABLE 6:** Computational time of the finite-element modeling workflow.

Segment	Geometry generation	FEM preparation	FE calculation
Time (min)	15.40	10.20	13.76

*Geometry-generation and FE-model-preparation times were measured for one representative case, whereas FE solution time represents the mean across 200 cases.

By comparison, the regression models required substantially less computational time after the simulation database had been established. As shown in Table 7, the training times of all models were below 0.65 s, while the recorded prediction times ranged from $1.558\times 10^{-4}$ to $2.490\times 10^{-2}$ s. For the final models selected in Section 3.4, the BPNN model for $E_{\text{c}}$ required 0.5771 s for training and 0.02490 s for prediction, whereas the SVR model for $\sigma_{\text{c}}$ required 0.3089 s for training and $4.109\times 10^{-4}$ s for prediction.

**TABLE 7:** Training and prediction times of the regression models.

Output	Models	Train time (s)	Predict time (s)
$E_{\text{c}}$	MLR	0.4683	0.001092
	QPR	0.02389	0.0005925
	SVR	0.6228	0.0003874
	Kriging	0.1070	0.002494
	BPNN	0.5711	0.02490
$\sigma_{\text{c}}$	MLR	0.01508	0.0002664
	QPR	0.006502	0.0001558
	SVR	0.3089	0.0004109
	Kriging	0.06771	0.0007453
	BPNN	0.6481	0.02018

These results demonstrate that the trained models can efficiently replace repeated finite-element simulations for parameter combinations within the sampled domain. The computational advantage is particularly relevant to large-scale parameter screening, response-surface generation, and iterative structural optimization.

### 3.6 Scope of application and limitations

This study integrates parametric Gyroid modeling, finite-element analysis, and machine learning to establish an efficient mapping from tissue-level and structural parameters to the apparent mechanical responses of cancellous-bone models. The controllable geometry, clearly defined input parameters, and uniformly sampled design space enable systematic investigation of the combined effects of $E_{\text{t}}$ , $\varepsilon_{\text{t}}$ , BV/TV , and DA . Furthermore, nested cross-validation and separate model selection for $E_{\text{c}}$ and $\sigma_{\text{c}}$ provide reliable estimates of predictive performance.

The regression models can predict finite-element responses within milliseconds, substantially reducing the time required for repeated geometry generation, meshing, and numerical solution. The framework is therefore well suited to parameter screening, response-surface analysis, sensitivity assessment, and iterative structural optimization. It also provides a computational basis for investigating how tissue properties and mesoscale architecture jointly determine the apparent stiffness and failure strength of porous bone models.

Nevertheless, the present study is based on an idealized Gyroid architecture and cannot fully represent the irregularity and heterogeneity of native cancellous bone. Future work could incorporate micro-CT-derived structures, tissue heterogeneity, and local defects associated with pathological bone changes into the modeling parameters, thereby improving the biological representativeness of the framework. Independent experimental data and anatomically specific datasets could also be introduced to evaluate the transferability of the models beyond the current parameter space.

Overall, the framework offers a rapid and reproducible approach for exploring the mechanical behavior of parametrically defined cancellous-bone structures. Future extensions combining experimental measurements, patient-specific imaging, and uncertainty quantification may further support individualized bone-mechanics analysis and biomimetic porous-structure design.

## 4 Conclusion

This study developed a rapid computational framework integrating parametric Gyroid modeling, finite-element analysis, and machine learning to predict the apparent mechanical properties of cancellous-bone models. The Gyroid finite-element model reproduced the overall force-displacement range reported in published compression experiments. Based on 200 parameter combinations generated by Latin hypercube sampling, five regression algorithms were evaluated using nested five-fold cross-validation to ensure consistent and leakage-free performance assessment.

The results demonstrated that the relationships between the tissue-level and structural parameters and the apparent mechanical responses were strongly nonlinear. BPNN provided the most accurate prediction of the apparent Young’s modulus, with an outer-fold $R^{2}$ of 0.9993 ± 0.0003, NRMSE of 0.0256 ± 0.0027, and MAPE of 1.68 ± 0.38%. SVR achieved the best performance for failure stress, with an $R^{2}$ of 0.9987 ± 0.0004, NRMSE of 0.0352 ± 0.0030, and MAPE of 2.23 ± 0.45%.

Response-surface analysis indicated that trabecular tissue Young’s modulus and bone volume fraction primarily governed apparent stiffness, whereas failure stress was additionally sensitive to tissue failure strain. Degree of anisotropy had a smaller but consistently positive effect on both responses. The observed nonlinear surfaces further demonstrated that the apparent mechanical behavior resulted from interactions between tissue properties and mesoscale architecture rather than from any single parameter alone.

After training, the selected models predicted finite-element responses within milliseconds, compared with approximately 39 min for geometry generation, finite-element model preparation, and numerical solution for one parameter combination. The proposed framework therefore provides an accurate, efficient, and reproducible tool for parameter screening, response-surface analysis, and structural design exploration within the investigated Gyroid model space. Future incorporation of micro-CT-derived architectures, tissue heterogeneity, local structural defects, and independent experimental data may further extend its applicability to anatomically specific and individualized bone-mechanics analyses.

## Data Availability

The original contributions presented in the study are included in the article/Supplementary Material, further inquiries can be directed to the corresponding author.
